# Can Water Rights Trading Scheme Promote Regional Water Conservation in China? Evidence from a Time-Varying DID Analysis

**DOI:** 10.3390/ijerph17186679

**Published:** 2020-09-14

**Authors:** Lin Fang, Fengping Wu

**Affiliations:** 1 Faculty of Mathematics and Physics, Huaiyin Institute of Technology, Huai’an 223001, China; 2 School of Business, Hohai University, Nanjing 210098, China; wfp@hhu.edu.cn

**Keywords:** water rights trading, regional water consumption, time-varying DID method, influence mechanism, heterogeneity analysis

## Abstract

Using the panel data of 30 provinces in China from 1998 to 2017, we adopt a time-varying difference-in-differences (time-varying DID) model to estimate the impact of water rights trading scheme on regional water consumption. The results show that water rights trading can significantly promote water conservation in the pilot regions by 3.1% compared to that in the non-pilot regions, and a series of robustness tests show consistent results. Policy effects are mainly driven by improving water-use efficiency and adjusting water structure; that is, by transferring water resources from the agricultural sector to the other sectors, agricultural water efficiency is improved and water conflict among sectors is alleviated; thus, water saving is achieved. In addition, by constructing two indexes of regional water pressure and tradable water resources, our heterogeneity analysis shows that water rights trading performs better in areas with high water pressure and large tradable water resources. Under the high pressure of large water use and low water endowment, water rights trading will evidently reduce water consumption more so than in the low-pressure regions, and with water rights trading, it is hard to achieve a policy effect in regions without sufficient tradable water resources. This paper provides important policy implications for China for further promoting the water rights trading scheme in the field of resource conservation.

## 1. Introduction

Water is a scarce and irreplaceable natural resource. China has a shortage of water resources; water resources per capita were 2354.92 m^3^ in 2016, being only one fourth of the world’s average level. In addition, the distribution of water resources exhibits significant differences among regions. For example, 80% of water resources are located south of the Yangtze River, while the northern regions have insufficient water resources, especially in the Huang-Huai-Hai Basin. Considering the gap between water endowment and water demand, with the pressure of increasing industrialization, urbanization and global climate change, water shortage has become one of the key factors restricting China’s sustainable development. In the pursuit of new-normal development in China, it is important to promote the reasonable conservation and recycling of water resources.

Since the 18th National Congress of the Communist Party of China (NCCPC), water rights trading scheme has been raised to be an important factor in supporting the construction of ecological civilization. Both the third and fifth plenary sessions of the 18th NCCPC proposed actively carrying out a pilot of water rights trading nationwide. In China, water resource management has gradually shifted to water-market construction; water rights trading has once again become one of the hot issues in the field of resources and the environment.

Some foreign countries have employed schemes of water rights trading to improve technology and save water. For instance, since an agreement was signed to trade water resources between San Diego city and California’s largest irrigation district in 2003, farmers were encouraged to save water by being paid an agreed price; thus, water conservation was achieved [1]. The case in Australia also showed that agricultural irrigation technology was improved since water rights trading was initiated [2]. Chile’s 1981 Water Code has been proved to realize Pareto optimization, so the World Bank and International Food Policy and Research Institute recommended that the Water Code was a successful mode for water management, particularly for developing countries [3]. “Water bank” in California has resulted in a large-scale redistribution of resources, improving regional water efficiency, promoting water conservation and achieving good social effects [4].

In China, many scholars believe that water rights trading can effectively improve allocation efficiency and thus promote water saving, compared with expanding the water supply by building water-conservancy projects. Water resources are transferred from entities with abundant endowment to those with poor endowment, and from low-benefit sectors to high-benefit sectors, thus improving water-use efficiency across the whole of society [5]. Liu et al. [6] and Zhang [7], respectively, verified the positive role in improving the efficiency of water-resource use, using the trading cases of Ningxia, Inner Mongolia, Guangzhou and Dongyang-Yiwu city.

Obviously, the current research on water rights trading in China mainly uses qualitative analysis and lacks systematic empirical testing. With the development of policy analysis tools such as “quasi-natural experiment”, difference-in-differences (DID) and its derivative methods have been used to evaluate policy effects in the field of resources and the environment. For example, Shen and Jin [8] and Hu et al. [9] studied the policy effect of a river-director system and carbon emission trading scheme based on DID and propensity scores matching-DID (PSM-DID), respectively. Shao et al. [10] studied the impact of an energy intensity constraint policy on total-factor energy efficiency based on DID and difference-in-difference-in-differences (DDD). Shi et al. [11] estimated the impact of smart-city construction on environmental pollution based on PSM-DID. Ren et al. [12] studied whether an emissions trading system improved a firm’s total-factor productivity based on DID and DDD. Considering that water rights trading is implemented randomly in some regions and not in others, in line with the research background of a “quasi-natural experiment”, therefore, this article uses a time-varying DID method to explore the water-saving effect of a water rights trading scheme.

In this paper, we adopt a time-varying DID model to estimate the impact of water rights trading scheme on regional water consumption, with the panel data of 30 provinces in China from 1998 to 2017. The results show that water rights trading can significantly promote water saving in the pilot regions by 3.1% compared to that in non-pilot regions, and a series of robustness tests show consistent results. Policy effects are mainly driven by improving water-use efficiency and adjusting water structure; that is, by transferring water resources from the agricultural sector to other sectors, agricultural water efficiency is improved and water conflict among sectors is alleviated; thus, water saving is achieved. In addition, by constructing two indexes of regional water pressure and tradable water resources, our heterogeneous analysis show that water rights trading performs better in the areas with high water pressure and large tradable water resources. Under the high pressure of large water consumption and low water endowment, water rights trading will evidently reduce water consumption more compared to in the low-pressure regions, and for water rights trading, it is hard to achieve a policy effect in the regions without sufficient tradable water resources as a basic aspect.

The main contributions of this paper are as follows. First, as far as we know, this is the first study to examine the water-saving effect of water rights trading scheme in China from a quantitative perspective. Second, against the background of water rights trading implemented in batches, lots of identification and robustness tests are conducted based on a time-varying DID method, thus effectively avoiding the endogeneity problem. Third, the potential influence mechanisms are investigated, and heterogeneous characteristics are revealed by constructing two indexes of regional water pressure and tradable water resources, so as to provide reasonable policy implications.

This paper is organized as follows. Section 2 describes the background of the water rights trading scheme. Section 3 presents a time-varying DID model and data. Section 4 describes the testing of the identified hypothesis. Section 5 proposes the baseline result and robustness test. Section 6 discusses the influence mechanism and heterogeneity analysis. Section 7 follows with the conclusion, and the final section presents policy implications.

## 2. Background of Water Rights Trading Scheme

According to the Interim Measures for the Administration of Water Rights Trading issued by the Ministry of Water Resources (2016) in China, water rights trading refers to water rights transferred among regions, basins, sectors and water-user associations driven by the market, on the basis of the reasonable definition and allocation of water rights. There are three types: regional water rights trading, inter-sector water rights trading and irrigation water users’ water rights trading. Regional water rights trading generally occurs between administrative regions in the same basin or within an inter-basin transfer project. Inter-sector water rights trading always take place between agriculture and industry. Industry invests and constructs the water-conveyance projects, and agriculture saves water that is transferred to the industrial sector by those water-conveyance projects. Irrigation water users’ water rights trading only happens in irrigation areas or organizations in the agricultural sector.

Water rights trading in China has been implemented in batches and promoted gradually. Since 2000, China has ushered in the first round of the pilot scheme (2000–2013), including regional trading (Dongyang and Yiwu cities in Zhejiang province), inter-sector trading (Ningxia and Inner Mongolia provinces) and irrigation water users’ trading (Zhangye city in Gansu province). From 2014 to 2017, China issued a series of documents on the construction of the water market, promoted the pilot of trading nationwide and initiated the second round, involving a total of 12 provinces in China. In 2014, there were nine pilot provinces, respectively, in Ningxia, Hubei, Inner Mongolia, Henan, Gansu, Guangdong, Jiangxi, Fujian and Xinjiang. In 2016, the National Water Rights Trading Platform was established; there were eight provinces that traded water rights, that is, Shanxi, Hebei, Beijing, Henan, Ningxia, Inner Mongolia, Xinjiang and Guangdong. Generally speaking, China’s water market has been promoted step by step, but the speed and depth of trading in the northern water-deficient regions are better than those in the southern water-abundant regions.

In the three types of water rights trading in China, most are regional and inter-sector trading; irrigation water users’ trading is progressing slowly and mainly occurs in a small number of irrigation areas such as Gansu, Hebei, Ningxia and Xinjiang. Regional trading includes three types; one is mainly conducted between different administrative regions in the same basin, such as Zhejiang and Fujian provinces (2003); Hebei, Shanxi and Beijing (2016); and the Dongjiang River Basin in Guangdong (2017); another is happening in inter-basin South–North water-transfer projects, such as those for Pingdingshan and Xinmi, and Nanyang and Xinzheng cities in Henan province (2016), and yet another is occurring in the same province, such as in Dongyang and Yiwu cities in Zhejiang (2000), and Quanzhou and Yongtai cities in Fujian (2014). At present, regional water rights trading is mainly represented as the consequence of political democratic consultation; the market mechanism does not play a big role.

Among them, inter-sector water rights trading performs well and has strong development prospects. For example, the trading of Ningxia and Inner Mongolia provinces in the upper and middle reaches of the Yellow River is a typical representative, making positive contributions to alleviating water contradiction and ensuring that regional water demand is met. Specifically, first, the local government provides essential water resources for new industrial projects and promotes rapid economic development without increasing total regional water consumption. For example, in Ordos city, 14 new industrial projects generate a benefit of CNY 26.6 billion per year after they receive traded water. Second, it expands the financing channels of water-conservancy projects, accelerates the construction of water-saving projects in irrigated areas, improves water-use efficiency and realizes the optimal allocation of water resources. Last, it protects farmers’ legitimate rights, reduces their losses in water conveyance and decreases their expenditure on water charges, thus benefiting the farmers. In this type, industry invests in the water-conveyance project, and water is saved by agriculture and transferred to industry. Thus, inter-sector water rights trading has explored a new path to ensure the sustainable development of water resources, that is, “agriculture supports industry and industry feeds agriculture”.

## 3. Methodology and Data

In the paper, we use a time-varying DID model to explore whether a water rights trading scheme promotes regional water conservation. The advantage of the DID model is that it can eliminate the impacts of macroenvironment and exogenous factors, thus obtaining reliable results by comparing the differences in water consumption before and after trading.

### 3.1. Time-Varying DID Model

The DID model has been widely used in the evaluation of policy effects; its basic principle is to construct the “treated group” with policy intervention and the “control group” without policy intervention. It compares the difference between the two groups before and after a policy shock to explain the policy’s effect. Considering that the implemented time for China’s water rights trading is different, this paper adopts the time-varying DID model [13,14] as follows:

(1) $$y_{it}=\alpha +\mu_{i}+\upsilon_{t}+\delta trading_{it}+\theta X_{it}+\varepsilon_{it}$$

In Equation (1), $y_{it}$ refers to the regional water consumption in year *t* of region *i*. $X_{it}$ represents a set of control variables capturing socioeconomic factors. $trading_{it}$ is a dummy variable that equals one in the years since region *i* has initiated trading and zero otherwise, according to Guo and Xiong [13] and Beck et al. [14]. Therefore, parameter δ captures the average impact of water rights trading on water consumption. A negative and significant δ indicates that water rights trading can reduce water use, while a positive and significant δ suggests that the water-saving effect is invalid. $\mu_{i}$ represents the individual fixed effect; it controls for the characteristics of regions that do not change over time, such as geographical conditions and natural endowments; $\upsilon_{t}$ represents the time-fixed effect, controlling for region-level shocks and trends over time such as macro-economic shocks, business cycles, and fiscal and monetary policies. $\varepsilon_{it}$ is the random error term, satisfying the zero mean and homoscedasticity hypothesis.

### 3.2. Variables and Data Description

This paper estimates the water-saving effect of a water rights trading scheme in China from 1998 to 2017. Our dataset only covers 30 provinces, excluding Tibet, Taiwan, Hong Kong and Macau because of missing data. The model includes a dependent variable (regional water consumption: *y_it_*), key independent variable (water rights trading scheme: *trading_it_*) and set of control variables *X_it_*.

According to Zhang et al. [15], the control variables should include economic scale, industrial structure, population scale and others. Thus, we use variables such as the per-capita GDP, ratio of added value of secondary and tertiary industries to GDP, and total population as control variables. In addition, per-capita water resources [16], the per-capita disposable income of urban residents [17] and regional chemical oxygen demand (COD) emission intensity (the ratio of regional COD emissions to GDP, referring to Xu and Cheng [18] and Zhang et al. [19]) are also added into control variables, respectively, representing the impact of natural conditions, income and environmental regulations.

For the index of water rights trading, we have manually sorted out the pilot timing of water rights trading in 30 provinces from 1998 to 2017 based on four data sources: the official documents published on the Baidu website, literature and news reports on China National Knowledge Infrastructure (CNKI), the report *analysis on water rights system construction in typical areas of Taihu Basin* issued by Hohai University and trading information on the website of the National Water Rights Trading Platform. According to the above information, we define the indicator *trading_it_* as a dummy variable that equals one in the years since region *i* has initiated trading and zero otherwise. We divide 30 provinces into pilot regions and non-pilot regions depending on the timing of water rights trading. The pilot regions include Zhejiang, Guangdong, Shanxi, Hebei, Jiangxi, Hubei, Henan, Xinjiang, Gansu, Ningxia, Inner Mongolia, Fujian and Beijing, which represent the “treated group” with different timing years, and the remaining 17 non-pilot provinces mean the “control group”. 

All of the data come from the *China Statistical Yearbook* and *China Environment Yearbook*. The monetary variables are adjusted to a constant 1998 price, and the descriptive statistical results are shown in Table 1.

## 4. Validity of the Identified Hypothesis

Our empirical analysis rests on two types of identified hypothesis. One is that the timing of water rights trading is unaffected by regional water consumption before trading. If water consumption directly affects the timing of water rights trading, the hypothesis of the exogenous treated group is violated; thus, we need to perform the test of exogenous choice for the treated group. The other is that the control group is a suitable counter-factual for the treated group, so we should perform the parallel-trend test.

### 4.1. Test of Exogenous Choice of Treated Group

According to Beck et al. [14], we explore whether the treated group is exogenously chosen by constructing the null hypothesis H0 and the alternative hypothesis H1 as follows:

**Hypothesis** **H0**. *There is no significant effect of regional water consumption before trading on the timing of water rights trading.*

**Hypothesis** **H1**. *There exists a significant effect of regional water consumption before trading on the timing of water rights trading.*

We use two methods to test the hypothesis. One is to draw a scatter plot of average regional water consumption prior to trading and the year of water rights trading. If there exists an evident positive or negative relationship between water consumption and the timing of water trading, then we accept the null hypothesis H0 and assert that the treated group satisfies the hypothesis of exogenous choice. The other method is to perform the regression of the year of water rights trading on the regional water consumption before trading. If the t-value of the coefficient is statistically significant, then we accept H0.

**Figure 1:**
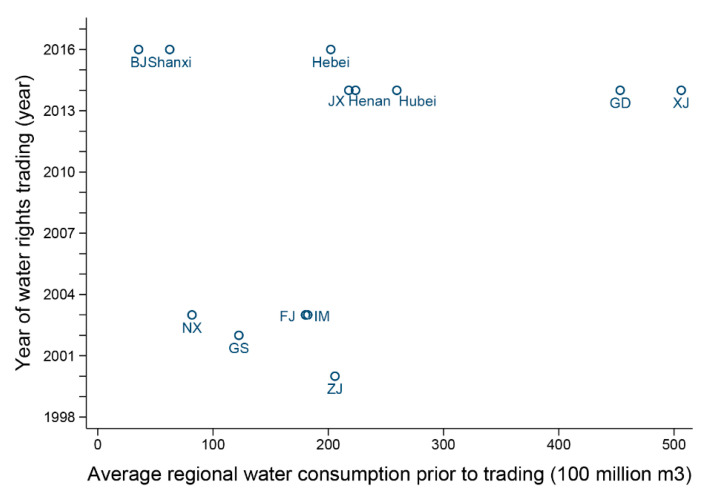
Timing of water rights trading and pre-existing regional water consumption.

As shown in the scatter plot Figure 1, the level of regional water consumption before trading does not explain the timing of water rights trading; there is no evident relationship between them. In addition, in the regression of the year of water rights trading on the regional water consumption before trading, we find the t-value is 1.1 and not significant. Both methods show that we should accept H0: the treated group is exogenously chosen.

Moreover, the timing of water rights trading in a region was decided by the central government and included in the national and local document plan as a mandatory regulation. Therefore, we believe that the problem of the endogeneity of choice of the treated group does not exist, and the hypothesis of an exogenously treated group is satisfied in our case.

### 4.2. Parallel-Trend Test

According to Beck et al. [14] and She et al. [20], a parallel trend means that there should be no trend difference between the treated group and control group in the pre-policy period. To prove the parallel trend, this paper interacts the treated variable with a series of dummy variables in the baseline Equation (1) to explore the pre-existing trend of the two groups as follows:

(2) $$y_{it}=\alpha +\beta_{-2}D_{it}^{-2}+\beta_{-1}D_{it}^{-1}+\beta_{0}D_{it}^{0}+\beta_{1}D_{it}^{1}+\ldots +\beta_{17}D_{it}^{17}+\theta X_{it}+\mu_{i}+\upsilon_{t}+\varepsilon_{it}$$

where $y_{it}$, $X_{it}$, $\mu_{i}$, $\upsilon_{t}$, and $\varepsilon_{it}$ represent the same as in Equation (1). $D_{s}^{\prime }$ are the dummy variables of water rights trading, and $D^{-j}$ equals one for region *i* in the *j*th year before the initiation of trading, while $D^{+j}$ equals one for region *i* in the *j*th year after the implementation of trading. For the timing year of trading, $D^{0}$ is equal to zero. In a 20-year window (1998–2017), we take the earliest initiation year 2000 as the baseline (Year 0), and all of the coefficients β represent the policy effect relative to Year 0. We construct the null hypothesis H0—$\beta_{-2}=\beta_{-1}=0$—and the alternative hypothesis H1: $\beta_{-2}\neq 0\;or\;\beta_{-1}\neq 0$. If the trend between the treated and control groups is the same, then the coefficients β before Year 0 should be insignificant, so we accept H0 and assert that the parallel-trend assumption is satisfied.

**Figure 2:**
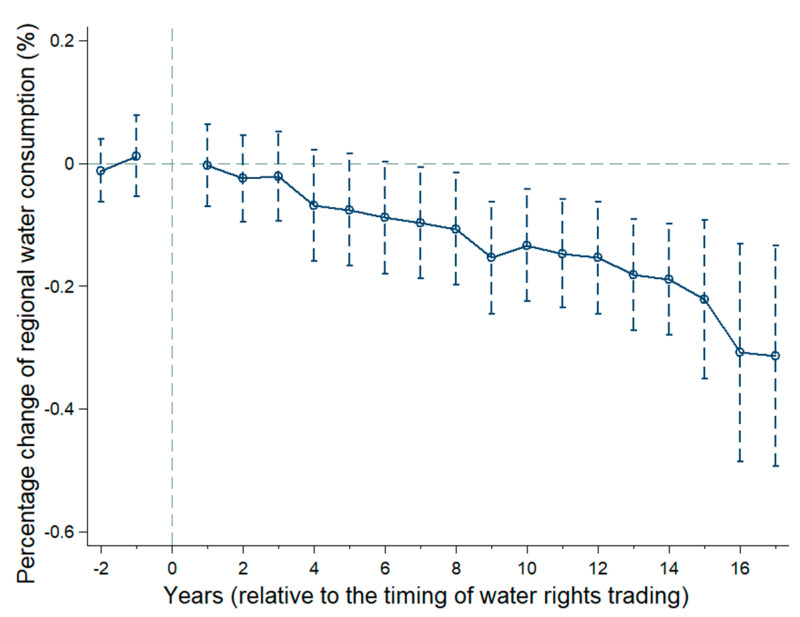
The dynamic impact of water rights trading on regional water consumption.

Figure 2 plots the estimation results and the 95% confidence intervals of the coefficients β, adjusted for region-level clustering. The dashed lines represent 95% confidence intervals, and circles in the middle of the dashed lines represent the estimated coefficients β of Equation (2).

The results illustrate two key points: the coefficients β are not statistically significant from zero during the pre-existing period, so we accept H0 and assert that the treated and control groups have a common trend prior to trading. The parallel assumption is satisfied. Additionally, the policy comes into effect in the seventh year after trading, indicating that water rights trading plays a positive role in water conservation, with a certain hysteresis effect.

## 5. Results and Robustness Test

### 5.1. Main Results

The results of the time-varying DID model are reported in Table 2. Column (1) without control variables shows that water rights trading significantly reduces regional water use (the coefficient is −0.046 and statistically significant). After gradually controlling for variables such as economic scale (column (2)), industrial structure (column (3)), population scale (column (4)), natural conditions (column (5)), environmental regulations (column (6)) and income (column (7)), we find that the results are still robust, with the negative and significant coefficient ranging from −0.042 to −0.027. In general, compared to that in the non-pilot regions, the total water consumption in the pilot regions will be reduced by 3.1% on average. The water-saving effect of the water rights trading scheme is evidently positive.

For the control variables, per-capita GDP and population will play a positive role in regional water conservation, and both exist in an inverted U-shaped relationship. It means that economic development and population growth will promote water demand in the short and medium term, and then, water demand will reduce gradually in the long term along with resource optimization and efficiency improvement. This is consistent with the result for industrial structure (the coefficient is −0.005 and statistically significant), which indicates that structure adjustment from agriculture to secondary and tertiary industries will improve the allocation and utilization efficiency for water, thus significantly reducing the total water use.

From the result for the COD emissions intensity, we find that the coefficient is 0.087 and statistically significant. Due to the negative relationship between water environmental regulation and COD emissions intensity, the result reveals that environmental regulation will indirectly reduce water consumption. With the dual goal of water conservation and emission reduction, the regulation of the water environment may be an effective path.

Additionally, there exists a U-shaped relationship between income and regional water consumption. That means urban residents save water at a low-income level, while they will not continue to save water at a high-income level. This phenomenon may be due to changes in water behavior and the willingness of residents. At the low-income level, residents will actively adjust home appliances from water-consuming to water-saving ones for saving money. However, at the high-income level, they tend not to care about the cost of water, leading to water waste. As a result, the total water consumption decreases first and then increases as income rises.

### 5.2. Robustness Test

To verify the reliability of the above conclusions, a series of robustness tests are conducted in this section. The results of these tests are shown in Table 3.

① Changing the index of water rights trading. In the three types of water rights trading, irrigation users’ trading is usually implemented in the small-scale irrigated regions and only initiated for a short period, differently from the other two types. Thus, we remove this type and re-construct a new index of water rights trading (described as Trading1) for regression. We can see that the result in column (1) is similar to the one presented before, implying that changing the core explanatory variable will not affect the estimated results.

② Deleting the data from 2008 to 2009. Declercq et al. [21], Bel and Joseph [22] thought that the 2008 economic crisis imposed a powerful effect on industrial enterprises and residents’ lives, thus affecting all kinds of resources. To eliminate the effect of the economic crisis on water resources, the data during this period are removed and the model is re-estimated. As shown in column (2), the result excluding the impact of the financial crisis is still robust.

③ Regression only with the data prior to 2014. The year 2014 was a turning point in the timing of water rights trading. Before that, water rights trading was mainly initiated by local governments independently, and the degree of marketization was relatively large. Since 2014, water rights trading has been gradually implemented at the national level. The country has set up seven pilot provinces and then established the National Water Rights Trading Platform for operating. Obviously, water rights trading should have greater a water-saving effect before 2014 compared to in the later years. Therefore, we exclude the years since 2014 and re-estimate. The result is shown in column (3); we find that the water-saving effect of water rights trading is still stable, with an average rate of 11.2%, implying that the marketization scheme has a significant effect.

④ Controlling the influence of the “three red lines” policy. In the field of water-resource management, the government has issued many policy guidelines and documents. The three red lines in 2012—the most stringent water-resource management system—is the most important one for controlling water quantity, water quality and water-use efficiency. In order to eliminate the policy effect, we expand the baseline Equation (1) with a dummy variable d2012 for the “three red lines” policy that equals 1 since the year 2012 and zero otherwise for any region. If the coefficient of trading is not significant, then the water-saving effect does not exist. As shown in column (4), due to the short time of policy implementation, the policy effect of the “three red lines” is not significant. However, the water-saving effect of water rights trading is still significant, which shows that the result is stable after excluding the influence of the “three red lines” policy.

⑤ Changing the dependent variable into agricultural water consumption. Agriculture has always been the key sector of water conservation, so we replace the original dependent variable (regional water consumption) with agricultural water consumption and re-estimate. The result is shown in column (5); we find that water rights trading also significantly reduces agricultural water use, with an average rate of 7.4%, which would promote regional water-saving and indicate that our results are robust.

## 6. Further Discussion

We have shown that the water rights trading scheme has a positive effect on water conservation. In this section, we explore the influence mechanism regarding water-structure adjustment and water-efficiency improvement. In addition, there are significant differences between water pressure and tradable water resources in the pilot regions; therefore, we study the heterogeneity characteristics of the water-saving effect by constructing the above two indexes with subgroup regression.

### 6.1. Mechanism Analysis

Water rights trading is a market-based scheme to transfer water rights among water users. Due to the imbalance in the distribution and utilization of water resources, the “invisible hand” will promote the trading of water rights among users driven by benefit, thus adjusting the regional water structure.

On the one hand, agricultural water use in China accounts for about 62% of the total water use; in Ningxia and Xinjiang, it is even as high as 90%. Agricultural irrigation technology is relatively low compared to the world’s advanced level; there is much space for efficiency improvement. On the other hand, deeper industrialization and urbanization increase water demand in the industrial sector, leading to water conflicts between agriculture and industry. Therefore, agricultural water will be the main body of water rights trading in China. Water trading from agriculture to other sectors can not only alleviate regional water conflicts among sectors and reduce water waste, but the agricultural sector, with high water endowment, will be also pushed to save water, thus realizing the coordination of water resources.

The influence of water-structure adjustment—agricultural water rights being transferred to other sectors—will realize water saving. Thus, we construct the model to explore whether China’s water rights trading scheme has realized the aim as follows:

(3) $$inddomagr_{it}=\alpha +\mu_{i}+\upsilon_{t}+\delta trading_{it}+\theta X_{it}+\varepsilon_{it}$$

where $X_{it}$, $trading_{it}$, $\mu_{i}$, $\upsilon_{t}$ and $\varepsilon_{it}$ represent the same as in Equation (1). *inddomagr_it_* refers to the ratio of industrial and domestic water to agricultural water (%), which measures the adjustment of water structure. The higher the value, the more the agricultural water that will be diverted to the secondary and tertiary industries, and the more reasonable the water structure will be. The coefficient δ represents the average impact of the water rights trading scheme on water structure. As shown in column (1) of Table 4, water rights trading will push agricultural water transfer to other sectors, with an average effect of 10.07% after controlling for the other variables, individual effects and time effects. Thus, the total water consumption is reduced due to the reasonable water-structure adjustment.

In addition, we want to know whether the water rights trading scheme has an impact on the water-use efficiency of both partners via water-structure adjustment; the models are constructed as follows:

(4) $$wueGDP_{it}=\alpha +\mu_{i}+\upsilon_{t}+\delta trading_{it}+\theta X_{it}+\varepsilon_{it}$$

(5) $$wueindustry_{it}=\alpha +\mu_{i}+\upsilon_{t}+\delta trading_{it}+\theta X_{it}+\varepsilon_{it}$$

(6) $${coeff}_{it}=\alpha +\mu_{i}+\upsilon_{t}+\delta trading_{it}+\theta X_{it}+\varepsilon_{it}$$

where *wueGDP_it_*, $wueindustry_{it}$ and ${coeff}_{it}$, respectively, represent regional water use per CNY 10,000 of GDP (m^3^), industrial water use per CNY 10,000 of industrial added value (m^3^) and the effective utilization coefficient for irrigation water (the data come from the *China Water Resources Bulletin* and the website of water-saving irrigation) (%). The first two variables measure water-use intensity in regions and industry, and the higher the value, the lower the water efficiency will be. The last variable measures the agricultural water-use efficiency, and the larger the value, the higher the water efficiency will be. The coefficient δ represents the average impact of the water rights trading scheme on water-use efficiency. It can be seen from the results in columns (2)–(4) of Table 4 that water rights trading causes water efficiency in agriculture and regions to increase by 1.2% and 3.8% on average, respectively. It shows that although there is no significant effect on industrial water efficiency, trading still actively prompts the improvement of water efficiency in the agricultural sector and the whole region, thus promoting regional water saving.

To sum up, water rights transfer from agriculture to other sectors alleviates water conflicts and realizes the structural optimization of water resources (Wang [23]). Moreover, it also improves the water-use efficiency of trading partners (Fang and Zhang [24]). Therefore, water rights trading has indeed produced a water-saving effect by the mechanisms of water-structure adjustment and water efficiency improvement.

### 6.2. Heterogeneity Analysis

As Tian and Hu [25] mentioned, the regions under high water pressure will actively promote the implementation of trading, so they will show a greater water-saving effect compared to the low-pressure regions. Besides, Han et al. [26] believe that the key point of the water market is to ensure sufficient tradable water resources, which depends on the capacity of the agricultural water rights supply. That is, the larger the agricultural water rights supply capacity, the more abundant the tradable water resources and the more successful the water market.

Considering the differences in water pressure and tradable water resources in the pilot regions, this paper verifies the heterogeneous characteristics regarding the two aspects. For the indicator of water pressure, we define it jointly by water supply and water demand, namely, a smaller supply and greater demand mean higher pressure. The indicator of tradable water resources is also defined in two parts: agricultural irrigation technology and the ratio of agricultural water, that is, lower technology and a higher ratio represent larger tradable water resources.

We adopt per-capita water endowment as the water supply, per-capita water consumption as the water demand, the effective utilization coefficient for irrigation water as irrigation technology, and the ratio of agricultural water to the total water as the ratio of agricultural water.

There is a negative relationship between per-capita water endowment and water pressure, the effective utilization coefficient and tradable water resources. There is a positive relationship between per-capita water consumption and water pressure, the ratio of agricultural water and tradable water resources. We standardize the negative indicators (per-capita water endowment and the effective utilization coefficient) and positive indicators (per-capita water consumption and ratio of agricultural water) as follows, respectively.

(7) $$index_{ij}^{-}=[index_{ij}-\min(index_{j})]/[\max(index_{j})-\min(index_{j})]$$

(8) $$index_{ij}^{+}=[\max(index_{j})-index_{ij}]/[\max(index_{j})-\min(index_{j})]$$

where $index_{ij}^{-}$ and $index_{ij}^{+}$ represent the standardized variables of the negative and positive indicators. 

Then, we take the weighted average (weight equals 0.5) of the above standardized variables as the comprehensive indicators to represent water pressure and tradable water resources, respectively.

According to the two indexes, we divide 30 provinces into two types: one type is low-pressure, moderate-pressure and high-pressure regions; the other type is small-tradable-water and large-tradable-water regions. As shown in column (1)–(3) of Table 5, we find that high-pressure and moderate-pressure regions both exhibit positive effects on water saving, while low-pressure regions do not. That means water rights trading only plays a role in relatively high-pressure regions and shows that the higher the pressure, the larger the water-saving effect.

The results in columns (4)–(5) show that with water rights trading, it is hard to achieve a water-saving effect in regions without sufficient tradable water resources. Therefore, it is necessary to reasonably increase the amount of tradable water in the region, so as to guarantee the maximal policy effect of water rights trading.

## 7. Conclusions

Water rights trading schemes have been raised by countries to play an important role in the field of resource conservation. One should consider that water rights trading in China is implemented randomly in some regions and not in others, in line with the research background of “quasi-natural experiment”. Therefore, this article uses a time-varying DID model to explore the water-saving effect of a water rights trading scheme based on the panel data of 30 provinces in China from 1998 to 2017. The results are as follows:

① A time-varying DID model without control variables and with control variables is estimated. Both results show that water rights trading can significantly promote water conservation. Compared to that in the non-pilot regions, the total water consumption in the pilot regions will be reduced by 3.1% on average.

② Robustness tests are conducted, including changing the index of water rights trading, eliminating the effect of the economic crisis, deleting some cases of trading, controlling the influence of the “three red lines” policy and changing the dependent variable. All of those robustness tests show consistent results.

③ The influence mechanisms are explored. Water rights trading promotes water-structure adjustment from agriculture to the other sectors, with an average effect of 10.07%. In addition, although there is no positive effect on industrial water efficiency, trading still actively causes water efficiency in agriculture and the whole region to increase by 1.2% and 3.8% on average, respectively. The water-saving effects are driven by the mechanisms of water-structure adjustment and water efficiency improvement.

④Heterogeneous characteristics are discovered. By constructing two standardized comprehensive indicators to represent water pressure and tradable water resources, we find that high-pressure and moderate-pressure regions both exhibit positive effects on water saving, while low-pressure regions do not. Compared to in the regions with large tradable water resources, with water rights trading, it is hard to achieve a policy effect in the regions without sufficient tradable water resources. In general, water rights trading performs better in areas with high water pressure and large tradable water resources.

## 8. Policy Implications

This paper provides important policy implications for China for further promoting a market-oriented water rights trading scheme in the field of resource conservation as follows:

Firstly, due to the positive effect of water rights trading, the government should continue to vigorously promote the pilot of trading nationwide, especially for the high-pressure regions with high water use and low water endowment such as Heilongjiang, Liaoning, Jiangsu and Anhui provinces.

Secondly, for the pilot regions, water-resource transfer from agriculture to the other sectors should be considered. Although this type of water-structure adjustment alleviates water conflicts and supports industrial development, we should particularly respect the legitimate rights of farmers and not incur any negative impact on agricultural production. Thus, the local government should pay special attention to the side-effects of water-structure adjustment. The government should guarantee that the basic demand of agricultural irrigated and acreage areas is met, besides compensating farmers if they suffer possible loss in agricultural production.

Thirdly, although water rights trading has a positive effect on agricultural and regional water efficiency, there is no active effect on industrial water efficiency. This phenomenon should raise concern. Water rights trading seems to be an action of “emergency”; water-deficient industry has only solved the problem of water shortage with the traded water, and water efficiency has not improved. The role of promoting transformation from water-consuming industry to water-conserving industry is not yet mature. Thus, the government should formulate a water-withdrawal permit system for industry and also implement a warning mechanism for the dual control of “water consumption” and “water efficiency” in industry.

Finally, we need to gradually improve tradable water resources in pilot regions. The key is to improve irrigation technology, using non-engineering measures instead of engineering measures. To be specific, the measures may include economic means—such as agricultural water pricing reform and eco-compensation-mechanism establishment, etc.—that can be used to effectively control the water-waste phenomenon. In addition, popularizing intelligent irrigation technology such as channel anti-seepage technology, pipeline water delivery, sprinkler irrigation, micro-irrigation, implementing crop precision irrigation, etc. should help to overcome the technology bottleneck and finally ensure the greatest water-saving effects of the water rights trading scheme.

This study can be continued in the future. First, our analysis is based on macro-data at the province level; the city, as the real entity of water rights trading, should have more interesting heterogeneity characteristics, and a possible research direction is to collect data at the city level and to perform estimation. Second, the control variables in the model can be further adjusted or expanded according to the new research. Third, the consumers’ perceptions of the supply of tap water could facilitate management by water companies and help to protect different groups of recipients that secure services of appropriate quality in the implementation of water rights trading; thus, the research perspective can be further expanded to the consumers’ perceptions of the supply of tap water [27]. Last, the “third-party effect” of water rights trading has been raised by some researchers [28,29], which refers to the fact that people or institutions other than the transferor and receiver of water rights may suffer losses or gain benefits in the process of trading. Therefore, we can estimate the third-party effect from the perspective of agricultural food safety and regional water pollution discharge.

## Figures and Tables

**Table 1:** Descriptive statistics of all variables.

Vector	Description (Unit)	Mean	StDev	Min	Max
y *	Regional water consumption (100 million m^3^)	191.9	134.4	16.4	591.3
trading	Water rights trading	0.2	0.4	0.0	1.0
PGDP *	Per capita GDP (yuan per person)	8175.2	4433.8	2326.5	25,206.0
structure	Ratio of added value of secondary and tertiary industries to GDP (%)	87.1	7.0	63.6	99.6
population *	Number of total population (10,000 person)	4365.1	2634.7	503.0	11,169.0
endowment *	Per capita water resource (m^3^ per person)	2161.6	2465.8	27.1	16,176.9
domincome *	Per capita disposable income of urban residents (yuan)	13,098.5	6979.6	4009.6	43,693.5
CODintensity *	Regional COD emission intensity (kg/10,000 yuan)	18.4	10.0	1.8	73.4

Notes: * data in logarithm form.

**Table 2:** The estimation results in the time-varying difference-in-differences (DID) model.

	Regional Water Consumption						
	(1)	(2)	(3)	(4)	(5)	(6)	(7)
trading	−0.046 ***	−0.042 ***	−0.040 **	−0.027 *	−0.027 *	−0.033 **	−0.031 *
	(−2.87)	(−2.65)	(−2.49)	(−1.66)	(−1.67)	(−2.09)	(−1.95)
PGDP		1.031 *	1.117 *	0.535	0.515	0.893	1.417 **
		(1.72)	(1.85)	(0.87)	(0.83)	(1.44)	(2.19)
PGDP2 ^#^		−0.062 *	−0.065 *	−0.0412	−0.04	−0.061 *	−0.089 **
		(−1.78)	(−1.87)	(−1.16)	(−1.12)	(−1.72)	(−2.40)
structure			−0.003	−0.004 *	−0.004 *	−0.004 *	−0.005 **
			(−1.14)	(−1.84)	(−1.85)	(−1.90)	(−2.13)
population				1.485 ***	1.498 ***	1.484 ***	1.645 ***
				(2.76)	(2.77)	(2.78)	(2.98)
pop2 ^#^				−0.121 ***	−0.122 ***	−0.118 ***	−0.135 ***
				(−3.37)	(−3.38)	(−3.31)	(−3.58)
endowment					−0.005	−0.005	−0.005
					(−0.33)	(−0.36)	(−0.39)
CODintensity						0.071 ***	0.087 ***
						(4.00)	(4.71)
domincome							−0.919 *
							(−1.91)
dominc2 ^#^							0.056 **
							(2.20)
constant	4.891 ***	0.628	0.326	−0.341	−0.268	−2.292	−1.133
	(315.39)	(0.24)	(0.13)	(−0.12)	(−0.09)	(−0.78)	(−0.33)
µ_i_	Yes	Yes	Yes	Yes	Yes	Yes	Yes
υ_t_	Yes	Yes	Yes	Yes	Yes	Yes	Yes
Obs	600	600	600	600	600	600	600
R^2^	0.279	0.284	0.286	0.317	0.317	0.336	0.346

Notes: t statistics in parentheses. *, ** and *** mean *p* < 0.10, *p* < 0.05 and *p* < 0.01, respectively. ^#^ represents the square term of the variable.

**Table 3:** Robustness check.

	Regional Water Consumption				Agricultural Water Consumption
	(1)	(2)	(3)	(4)	(5)
trading		−0.031 *	−0.112 ***	−0.031 *	−0.074 ***
		(−1.87)	(−5.18)	(−1.95)	(−3.90)
trading1	−0.030 *				
	(−1.83)				
d2012				0.179	
				(1.63)	
Constant	−1.088	−0.905	0.656	−1.133	−5.603
	(−0.31)	(−0.25)	(0.16)	(−0.33)	(−1.35)
Control variables	Yes	Yes	Yes	Yes	Yes
µ_i_	Yes	Yes	Yes	Yes	Yes
υ_t_	Yes	Yes	Yes	Yes	Yes
Obs	600	540	480	600	600
R^2^	0.346	0.353	0.435	0.346	0.362

Notes: t statistics in parentheses. *, ** and *** mean *p* < 0.10, *p* < 0.05 and *p* < 0.01, respectively.

**Table 4:** The results for the influence mechanisms.

	Water Structure	Agricultural Water Efficiency	Industrial Water-Use Intensity	Total Water-Use Intensity
	(1)	(2)	(3)	(4)
trading	10.071 ** (2.40)	0.012 ** (2.01)	0.008 (0.24)	−0.038 ** (−2.25)
Constant	1300 (1.46)	−1.032 (−0.77)	23.653 *** (3.08)	15.368 *** (4.20)
Control variables	Yes	Yes	Yes	Yes
µ_i_	Yes	Yes	Yes	Yes
υ_t_	Yes	Yes	Yes	Yes
Obs	600	600	600	600
R^2^	0.390	0.767	0.346	0.744

Notes: t statistics in parentheses. *, ** and *** mean *p* < 0.10, *p* < 0.05 and *p* < 0.01, respectively.

**Table 5:** The result of heterogeneity analysis.

	Low-Pressure	Moderate-Pressure	High-Pressure	Small Tradable Water	Large Tradable Water
	(1)	(2)	(3)	(4)	(5)
trading	−0.005 (−0.19)	−0.044 * (−1.67)	−0.096 *** (−3.22)	0.014 (0.56)	−0.068 *** (−4.03)
Constant	56.986 *** (8.12)	−13.625 ** (−2.10)	13.841 * (1.83)	−33.414 *** (−6.07)	21.916 *** (4.31)
Control variables	Yes	Yes	Yes	Yes	Yes
µ_i_	Yes	Yes	Yes	Yes	Yes
υ_t_	Yes	Yes	Yes	Yes	Yes
Obs	200	200	200	300	300
R^2^	0.694	0.558	0.575	0.511	0.515

Notes: t statistics in parentheses. *, ** and *** mean *p* < 0.10, *p* < 0.05 and *p* < 0.01, respectively.
